# The Rhizobacterium *Bacillus amyloliquefaciens* subsp. *plantarum* NAU-B3 Contains a Large Inversion within the Central Portion of the Genome

**DOI:** 10.1128/genomeA.00941-13

**Published:** 2013-11-14

**Authors:** Huijun Wu, Junqing Qiao, Jochen Blom, Christian Rueckert, Oleg Reva, Xuewen Gao, Rainer Borriss

**Affiliations:** Department of Plant Pathology, College of Plant Protection, Nanjing Agricultural University, Key Laboratory of Monitoring and Management of Crop Diseases and Pest Insects, Ministry of Agriculture, Nanjing, Chinaa; Institute of Plant Protection, Jiangsu Academy of Agricultural Sciences, Nanjing, Chinab; Computational Genomics, Center for Biotechnology (CeBiTec), Universität Bielefeld, Bielefeld, Germanyc; Department of Biochemistry, Bioinformatics and Computational Biology Unit, University of Pretoria, Hillcrest, Pretoria, South Africad; Institut für Biologie, Humboldt-Universität Berlin, Berlin, Germanye; ABiTEP GmbH, Berlin, Germanyf

## Abstract

The genome of rhizobacterium *Bacillus amyloliquefaciens* subsp. *plantarum* strain NAU-B3 is 4,196,170 bp in size and harbors 4,001 genes. Nine giant gene clusters are dedicated to the nonribosomal synthesis of antimicrobial lipopeptides and polyketides. Remarkably, NAU_B3 contains a large inversion within the central portion of the genome.

## GENOME ANNOUNCEMENT

The aerobic, endospore-forming rhizobacteria belonging to *Bacillus amyloliquefaciens* subsp. *plantarum* are known for enhancing the yields of crop plants and for suppressing microbial plant pathogens (1, 2). The type strain, *B. amyloliquefaciens* FZB42, was shown to colonize plant roots (3), synthesize antimicrobial secondary metabolites (4–8), and produce plant growth-promoting compounds, such as indole-3-acetic acid (IAA) (9), and volatile compounds (1). Recently, several representatives of the plant-associated *B. amyloliquefaciens* subsp. *plantarum* group have been sequenced (5, 10–13), allowing for comparative genomics of this important group of plant growth-promoting bacteria. Here, we report the genome sequence of the plant-associated strain NAU-B3.

Strain NAU-B3, isolated from the wheat rhizosphere in Jiangsu province, east China, was identified as being from *B. amyloliquefaciens* subsp. *plantarum* (2). The strain contains a cryptic 8,438-bp plasmid pBSG3 harboring the RapQ/PhrQ system (14).

Genomic DNA prepared from NAU-B3 was used for the construction of a 3-kb long paired-end library with a GS FLX library preparation kit (Roche, Mannheim, Germany) in combination with GS FLX paired-end adaptors (Roche), according to the manufacturer’s protocol. The reads were assembled using the GS *de novo* Assembler and the resulting scaffolds were oriented based on the occurrence of unique single nucleotide polymorphisms (SNPs) in the repetitive rRNA (RRN) contigs. Utilization of the paired-end information allowed for the scaffolding of contigs that are >500 bp. Gap closure was done by long-range PCR (Phusion polymerase; New England BioLabs, Frankfurt [Main], Germany) and subsequent Sanger sequencing (IIT Biotech, Bielefeld, Germany). The prediction of protein-coding sequences was initially accomplished with REGANOR (15). Manual and automatic annotation was done using the annotation software GenDB 2.4 (16).

The complete genome sequence of NAU B3 consists of a circular 4,196,170-bp chromosome with a G+C value of 45.99%. The genome was larger than that of FZB42 due to many phage insertions not present in FZB42. The chromosome consists of 4,001 genes, 10 rRNA operons, and 92 tRNAs.

Nine gene clusters, covering 8.5% of the whole genome, were involved in nonribosomal synthesis of lipopeptides, such as surfactin, bacillomycin D, and fengycin, the siderophore bacillibactin, an *nrpS* gene cluster with unknown function, the polyketides bacillaene, difficidin, and macrolactin, and the dipeptide bacilysin. Notably, a giant genome inversion took place within the *malS* gene at position 924442. This event resulted in separating the C-terminal from the N-terminal portion of *malS* by a 2.11-Mb insertion.

### Nucleotide sequence accession numbers.

The complete sequences of NAU-B3 have been deposited in EMBL (accession no. for the complete genome, HG514499.1, and for plasmid pBamNAU-B3a, HG514500.1).
